# Effect of air-particle abrasion and methacryloyloxydecyl phosphate primer on fracture load of thin zirconia crowns: an *in vitro* study

**DOI:** 10.3389/fdmed.2024.1501909

**Published:** 2024-11-25

**Authors:** Ruwaida Z. Alshali, Mohammed A. Alqahtani, Basem N. Alturki, Loay I. Algizani, Abdullah O. Batarfi, Zuhair K. Alshamrani, Razan M. Faden, Dalea M. Bukhary, Mosa M. Altassan

**Affiliations:** ^1^ Oral and Maxillofacial Prosthodontics Department, Faculty of Dentistry, King Abdulaziz University, Jeddah, Saudi Arabia; ^2^ Prosthetic Dental Science, College of Dentistry, King Saud University, Riyadh, Saudi Arabia; ^3^ Dental Department, King Faisal Hospital-Makkah, Ministry of Health, Riyadh, Saudi Arabia; ^4^ Oral and Maxillofacial Prosthodontics Department, King Abdulaziz University Dental Hospital, Jeddah, Saudi Arabia; ^5^ Taif Specialized Dental Center, Ministry of Health, Riyadh, Saudi Arabia; ^6^ Faculty of Dentistry, King Abdulaziz University, Jeddah, Saudi Arabia; ^7^ Al Qurayyat Dental Center, Ministry of Health, Riyadh, Saudi Arabia

**Keywords:** zirconia, crowns, fracture, strength, air abrasion, 3Y-TZP, 5Y-PSZ, 10-MDP primer

## Abstract

The study aimed to investigate the effects of airborne-particle abrasion (APA) and 10-methacryloxydecyl dihydrogen phosphate (10-MDP primer) surface treatments on the fracture load of thin zirconia crowns made from 3Y-TZP and 5Y-PSZ zirconia. Eighty full-contour zirconia crowns of 0.5 mm thickness were fabricated from 3Y-TZP and 5Y-PSZ zirconia. Crowns of each material were divided into four groups based on the surface treatment applied to the fitting surface (*n* = 10): Group 1 (control), Group 2 (10-MDP Primer Only), Group 3 (APA Only), and Group 4 (10-MDP Primer + APA). Crowns were cemented using self-adhesive resin cement and subjected to thermocycling. Fracture load tests were performed using a universal testing machine with a hemispherical indenter. Statistical analysis was performed using one-way ANOVA, Tukey's *post hoc* test, and independent samples *T*-test (*α* = 0.05). The fracture load of 3Y-TZP was significantly higher than 5Y-PSZ crowns across all groups (*P* ≤ 0.001). Group 1 had the lowest fracture load, while Group 4 had the highest for both materials. In 3Y-TZP, the fracture load of Group 2 increased by 40% (*P* = 0.002) and Group 3 by 50% (*P* < 0.001) compared to Group 1. Group 4 showed a 90% increase over Group 1 (*P* < 0.001). For 5Y-PSZ, fracture load of Group 4 increased by 70% compared to Group 1 (*P* < 0.001). It was concluded that applying a 10-MDP primer or APA significantly increases the fracture load of thin 3Y-TZP zirconia crowns, with the combination of both treatments yielding the highest values. For 5Y-PSZ, a significant increase in fracture load is observed only when both APA and the 10-MDP primer are used together.

## 1 Introduction

Zirconia crowns have gained popularity in modern dentistry due to their superior mechanical properties, wear resistance, aesthetics, and enhanced tooth color matching. Their fabrication is further facilitated by digital techniques, making them widely used (1). Dental zirconia is composed of zirconium oxide, which exists in three crystalline phases depending on temperature: monoclinic, tetragonal, and cubic. At room temperature, zirconia is monoclinic, brittle, and unsuitable for dental use. However, when stabilized with 3 mol% yttrium oxide (forming 3Y-TZP), it retains its tetragonal structure at room temperature, which is preferred for its high fracture toughness (3.5 to 4.5 MPa·m¹/²) and flexural strength (1,200–1,500 MPa) (1). This toughness is due to phase transformation toughening, where stress induces a transformation from the tetragonal to monoclinic phase, causing volume expansion that generates compressive stresses to inhibit crack propagation (2). In contrast, at temperatures above 2,370°C, zirconia adopts the cubic phase, which, while more translucent, lacks the mechanical benefits of phase transformation toughening (3).

The first-generation zirconia has high opacity due to the asymmetrical arrangement of tetragonal crystals, making it suitable primarily as a framework material veneered with more esthetic materials (4).The second-generation zirconia improved translucency by reducing aluminum oxide content which acts as a sintering aid from 0.25 wt% to 0.05 wt% (5). Although the improvement in aesthetics was modest, it was still not suitable for anterior teeth without veneering (6), but it maintained the high fracture toughness and flexural strength similar to the first generation (7). The third-generation zirconia increased yttrium oxide content to 5 mol% (5Y-PSZ), resulting in more than 50% cubic phase (3). This change significantly improved translucency, however, the mechanical properties such as fracture toughness were reduced due to the lack of phase transformation toughening (3). Third-generation zirconia was modified by decreasing the content of yttrium oxide from 5 mol% to 4 mol% (4Y-PSZ), balancing mechanical properties and aesthetics. It has more than 25% cubic phase, offering better translucency than first- and second-generation zirconia while retaining higher mechanical fracture load compared to 5Y-PSZ (1).

The thickness of zirconia crowns plays a crucial role in their fracture strength. Based on *in vitro* studies assessing fracture load of zirconia crowns of different thicknesses, the recommended and minimum thicknesses for zirconia crowns vary depending on the material used and the expected occlusal loads (7–11). A minimum occlusal thickness of 1 mm is usually advised to ensure the material can withstand typical and higher occlusal forces. In situations with limited interocclusal space or teeth with short clinical crowns, thin zirconia crowns are often required to preserve dental tissues. Studies have shown that at reduced thicknesses, 3Y-TZP zirconia crowns can maintain adequate fracture load with occlusal thickness as low as 0.5 mm, making them viable for clinical use in space-constrained situations (12, 13).

Surface treatments are essential for improving the bond strength between resin cements and zirconia, which in turn improves the fracture load of the restorations (8). Enhancing bond strength helps create a more integrated restoration, reducing the likelihood of de-bonding and increasing overall durability (14). Various surface treatments include airborne-particle abrasion (APA), tribochemical silica coating (TBS), hot chemical etching, plasma treatment, and selective infiltration etching (SIE) (15). APA increases surface roughness and wettability for better mechanical retention (16, 17). Combining airborne-particle abrasion (APA) with 10-methacryloyloxydecyl dihydrogen phosphate (10-MDP) primers significantly enhances bond strength by forming durable phosphorus-oxygen-zirconium bonds with zirconia. Studies show that this combination results in higher shear bond strength compared to non-primed groups, making it the gold standard for bonding zirconia restorations (18, 19). The improvement is especially notable when the resin cement does not contain MDP (20), though bond strength is enhanced regardless of the cement type (21). The impact of APA on flexural strength varies by zirconia generation: in 3Y-TZP, APA with 110 μm alumina particles at 0.4 MPa for 15 s improves flexural strength through phase transformation toughening (22). In contrast, for 4Y-TZP and 5Y-PSZ, increasing APA pressure beyond 0.2 MPa introduces microcracks that reduce flexural strength. Therefore, a pressure of around 0.2 MPa and 30–50 μm particles is optimal for balancing bond strength and flexural strength in these newer materials (23, 24).

No previous studies were performed assessing the fracture load of thin zirconia crowns (0.5 mm thickness) comparing 3Y-TZP and 5Y-PSZ bonded zirconia in combination with different surface treatment methods. The current *in vitro* study aims at assessing the impact of different treatments of the intaglio surface on the fracture load of thin monolithic zirconia crowns. The different surface treatments involve the use of 10-MDP primer and APA either individually or in combination to determine how these treatments would influence the fracture load of thin zirconia crowns fabricated from both 3Y-TZP and 5Y-PSZ zirconia materials. The main null hypotheses to be tested are that the type of zirconia material has no effect on the fracture load of thin zirconia crowns, and that the use of 10-MDP primer and APA surface treatments, either individually or in combination, has no effect on the fracture load of the crowns. The results of this study would help in determining the importance, the draw backs, or the unnecessity for the use of any of these protocols in terms of the fracture load of thin zirconia crowns especially for the 5Y-PSZ recent generation zirconia material.

## 2 Materials and methods

### 2.1 Tooth preparation

This research received ethical clearance through the Scientific Research Ethics Committee within the Faculty of Dentistry at King Abdulaziz University (Reference No. 213-01-21). A total of 80 healthy human premolar teeth, extracted for orthodontic purposes, were gathered and sterilized using 5% sodium hypochlorite for 10 min before being preserved in normal saline with pH of 4.5–7.0. Teeth that had caries or previous dental work were excluded; only intact teeth with consistent bucco-lingual, mesio-distal, and corono-apical measurements (±1 mm) were chosen. An ultrasonic scaler was used to eliminate external debris, dental plaque, and calculus. To replicate conditions of the oral cavity, these teeth were kept in an incubator at 37°C with 90% humidity level (General purpose incubator 6T2-2, Sheldon, US). The roots of the teeth were dipped into molten carving wax to a depth of 2 mm beneath the cementoenamel junction (CEJ). A cold-cure acrylic resin (ECO-ACRYL COLD, protechno, 17469 VILAMALLA, Girona, Spain) was then prepared to a fluid consistency and poured into a mold where the roots were embedded to mimic the alveolar bone. Once the resin had fully hardened, the blocks were immersed in warm water to remove any wax residue, effectively creating a consistent gap between the tooth roots and the acrylic mold, akin to the space of a periodontal ligament. Low-viscosity vinylpolysiloxane silicone material (Variotime light flow, Kulzer GmbH, Hanau, Germany) was injected into the acrylic sockets before carefully positioning the roots, creating a thin silicone layer around each root simulating periodontal ligaments as described in a previous study (25).

### 2.2 Fabrication of zirconia crowns

Teeth were prepared using the following protocol: a 0.5 mm finish line width with chamfer configuration positioned coronal to the cementoenamel junction (CEJ) by 1 mm, axial reductions of 0.5–0.7 mm, and occlusal reduction of 1 mm. A single operator performed all preparations using a high-speed, coarse tapered diamond bur equipped with a guidance axial pin to regulate the width of the chamfer finish line, while employing water cooling for temperature control (Bur number 508 534 016, FG, Meisinger, Germany). Preparations were digitally imaged (Canon EOS 1200D DSLR camera) and their dimensions were quantified using ImageJ software (ImageJ 1.53t, Wayne Rasband and contributors, National Institutes of Health, USA). The measured dimensions were a convergence angle of 17.8° (±3.5) mesiodistally and 17.6° (±5.6) buccolingually, an occlusogingival height of 4.1 mm (±0.7), a mesiodistal width of 5.6 mm (±1.0), and a buccolingual width of 8.0 mm (±1.0).

A total of 80 full-contour zirconia crowns were fabricated using two different types of zirconia. Forty crowns were made from 3Y-TZP high-translucency zirconia (Cercon ht) and forty from 5Y-PSZ extra-high-translucency zirconia (Cercon xt). The material composition and manufacturer information are detailed in Table 1.

**Table 1:** Test materials’ composition and manufacturer details.

Material	Composition (wt%)	Manufacturer	Lot number/shade
Cercon ht (high translucent zirconia), 2nd generation zirconia (3Y-TZP)^a^	– Zirconium oxide (ZrO_2_) – 5% Yttrium oxide (Y_2_O_3_), (3 mol%) – <3% Hafnium oxide – <1% aluminium oxide, silicon oxide, and other oxides	Dentsply Sirona, Charlotte, North Carolina, USA	18042814/Shade B1
Cercon xt (extra translucent zirconia), 3rd generation zirconia (5Y-PSZ)^b^	– Zirconium oxide (ZrO_2_) – 9% Yttrium oxide (Y_2_O_3_) (5 mol%) – <3% Hafnium oxide – <1% aluminium oxide, silicon oxide, and other oxides	Dentsply Sirona, Charlotte, North Carolina, USA	18043227/Shade B1
Multilink Speed (self-adhesive self-curing dental resin cement with light curing option)	– Monomer matrix: dimethacrylates and acidic monomers (10-MDP monomer)^c^ – Inorganic fillers (40 vol%): barium glass, ytterbium trifluoride, co-polymer, highly dispersed silicon dioxide. Size of fillers ranges from 0.1 µm to 7 µm (mean particle size of 5 µm – Initiators, stabilizers, and color pigments (<1%)	Ivoclar vivadent AG, 9494 Schaan, Liechtenstein	Z02HXH/Transparent
Monobond N	– Alcohol solution of silane methacrylate – Phosphoric acid methacrylate (10-MDP monomer) – sulphide methacrylate	Ivoclar vivadent AG, 9494 Schaan, Liechtenstein	Z02XRS

^a^ 3 mol% yttria-stabilized tetragonal zirconia polycrystals.

^b^ 5 mol% yttria partially stabilized zirconia.

^c^ 10-methacryloxydecyl dihydrogen phosphate.

**Figure 1:**
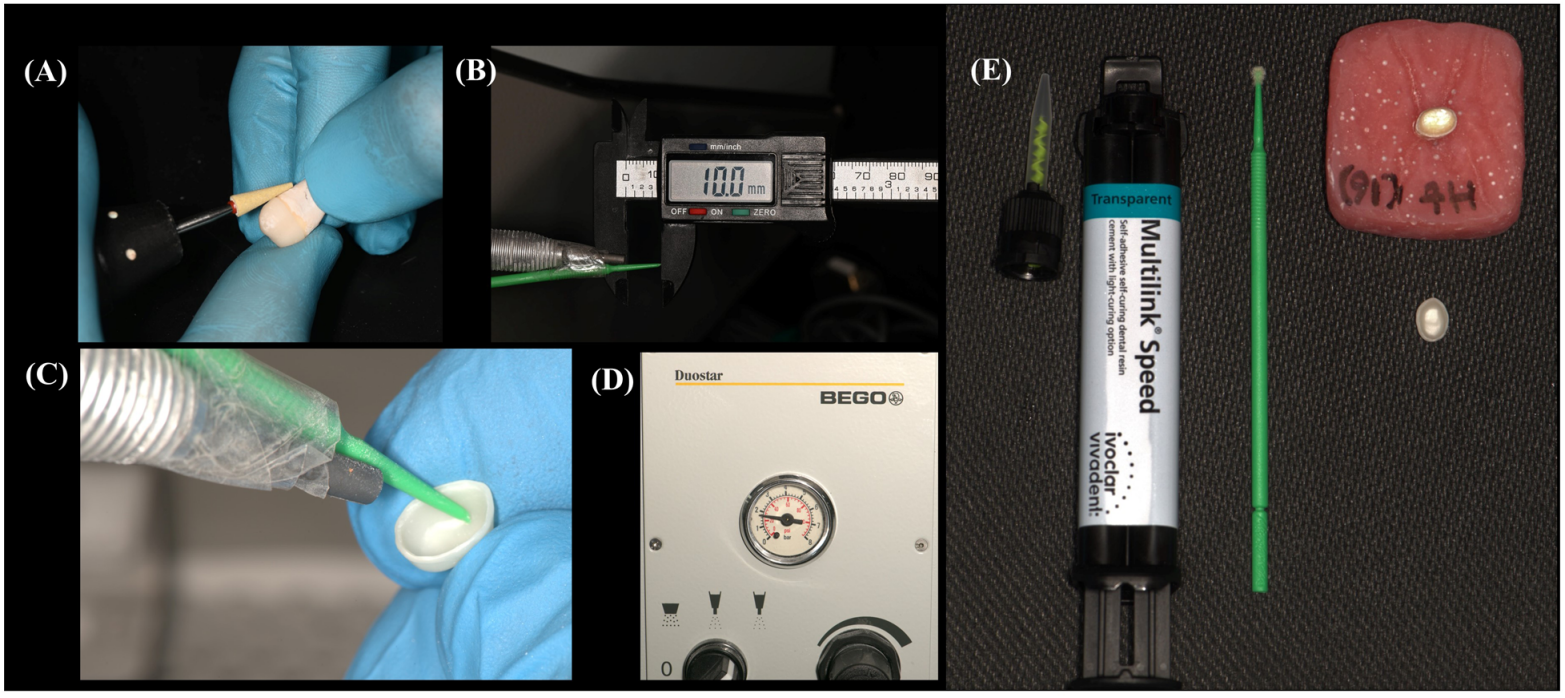
Images illustrating the preparation and cementation process of a thin monolithic zirconia crown: **(A)** polishing the crown using a diamond polishing bur. **(B,C)** A plastic jig fitted at the air abrasion tip ensures a consistent 10-mm distance between the air abrasion handpiece tip and the fitting surface of the crown. **(D)** The air abrasion device set at a pressure of 25 psi (0.17 MPa). **(E)** The crown, prepared for cementation onto its corresponding tooth, with self-adhesive resin cement applied through an auto-mixing tip.

Before scanning, the preparations were coated with Scantist 3D Vanishing Spray (Scantist 3D, Johann-Strauss-Str. 13, 45657 Recklinghausen, Germany). The impressions were captured using a Ceramill Map 600 scanner (Amann Girrbach, Gewerbestraße 10, 6841 Mäder, Austria), and the designs for the crowns were developed using Ceramill Mind software. These designs featured a non-anatomic full-contour profile with a uniform thickness of 0.5 mm and a 50 µm cement spacer. The crowns were then milled using Ceramill Motion 2 (Amann Girrbach, Gewerbestraße 10, 6841 Mäder, Austria) and sintered in an InFire HTC Speed furnace (Dentsply Sirona, Charlotte, North Carolina, USA) following a conventional slow sintering protocol (peak temperature: 1,520°C, dwell time: 130 min, heating rate: 11–31°C/min, cooling rate: 22°C/min). Finally, a single operator carried out the final finishing and polishing using a finishing kit with various grit sizes diamond polishing burs (TwisTec Celtra Set, Dentsply Sirona, Charlotte, North Carolina, USA) (Figure 1A).

### 2.3 Cementation procedure

The crowns of each material were split into four groups (*n* = 10) according to the treatment of their intaglio surfaces:

– Group 1 (Control) received no surface treatment; the crowns were only cleaned with Ivoclean (Ivoclar Vivadent, AG, 9494 Schaan, Liechtenstein) for 20 s, rinsed thoroughly with water spray, and dried with grease-free air.
– Group 2 (10-MDP Primer Only) was cleaned with Ivoclean and dried, then treated with a universal primer containing 10-MDP monomer (Monobond N, Ivoclar Vivadent AG, 9494 Schaan, Liechtenstein) applied in a single layer to the intaglio surface using a micro-brush. The primer was allowed 50 s to react, and any excess monomer was dispersed with air.
– Group 3 (APA Only) had their intaglio surfaces subjected to APA using 50 µm aluminum oxide particles (Korox, BEGO GmbH & Co. KG, Wilhelm-Herbst-Straße 1, 28359 Bremen, Germany) at a 10 mm distance and 0.17 MPa pressure for 5 s. The distance was maintained using a fixed object fitted on the air abrasion handle (BEGO duoster z blaster, Germany) (Figures 1B–D). After APA, the crowns underwent ultrasonic cleaning in distilled water for 5 min, followed by cleaning with Ivoclean, rinsing, and drying.
– Group 4 (10-MDP Primer + APA) followed the same procedure as Group 3 for APA and cleaning, and then received an additional treatment with 10-MDP primer as described in Group 2.

All crowns were cemented using Multilink Speed self-adhesive dual-cure resin cement containing 10-MDP (Ivoclar Vivadent AG, 9494 Schaan, Liechtenstein). Before cementation, each tooth was cleaned of scanning powder with pumice and a prophy brush using a low-speed handpiece, completely rinsed with water, and dried with grease-free air. The cement was applied through an auto-mixing tip (Figure 1E), and the crowns were firmly seated on the prepared teeth. Excess cement was removed with a micro-brush, and the crown margins were protected from oxygen inhibition by immediately covering them with a glycerin gel. The crowns were then placed in a universal mechanical testing machine using a static load of 5 N to apply pressure on the crowns for 5 min (Instron, model 5944, 825 University Ave, Norwood, MA, 02062-2643, US). This was followed by light curing of all cement joints for 20 s on each surface using an LED light curing unit (Demi Ultra, Kerr, Orange, California, USA) with an irradiance of approximately 1,200 mW/cm^2^ and a wavelength range of 450–470 nm. Subsequently, the glycerin gel was rinsed off with water, and the restoration margins were polished using a zirconia finishing kit (TwisTec Celtra Set, Dentsply Sirona, Charlotte, North Carolina, USA).

### 2.4 Thermocycling (hydrothermal aging)

Following a 24-hour storage period at room temperature, the cemented crowns were subjected to a thermocycling regimen to simulate hydrothermal aging. This process involved 10,000 cycles (simulating one year of intraoral thermal stresses) using SD Mechatronik Thermocycler equipment (Westerham, Germany), alternating between two baths at temperatures of 5°C and 55°C. The dwell time between each temperature immersion was 10 s. This process simulates one year of intraoral thermal stresses. Prior to mechanical testing, all samples underwent this thermocycling sequence.

### 2.5 Load-to-failure test

The fracture load of the crowns was assessed using a universal mechanical testing machine equipped with a 10 kN load cell (Instron, model 5944, 825 University Ave, Norwood, MA, 02062-2643, US). A custom-made hemispherical stainless-steel indenter (diameter = 6 mm) was accurately positioned at the central fossa of the occlusal surface for each crown. To prevent contact damage, a piece of an extra heavy rubber dam sheet (0.5 mm thickness) was placed between the indenter and the crown (roeko Flexi Dam non latex, REF 390 035, COLTENE, 89129 Langenau, Germany). Each crown was first subjected to a vertical preload of 20 N, then compressively loaded at a crosshead speed of 0.5 mm/min until fracture occurred. Fracture was identified by a sudden, sharp drop in the load/displacement curve. Fractography analysis of the broken crowns was conducted visually using ×6 magnifying loupes, classifying fractures into five types based on fracture patterns identified in a previous study (26). Type I involves a cohesive cervical fracture or crack near the finish line. Type II is characterized by a cohesive fracture within the crown that does not involve the interface. Type III shows a fracture at the interface, with the core remaining intact. Type IV features a fracture that involves the core but preserves the root. Finally, Type V is the most severe, with the fracture extending through the crown, core, and into the root. Each type of fracture was identified and documented by the same operator.

### 2.6 Statistical analysis

To ensure sufficient statistical power for detecting significant effects in the study, a sample size estimation was performed using G*Power software version 3.1.9.7 (University of Düsseldorf, Düsseldorf, Germany). The analysis was based on the following parameters: alpha error probability of 0.05, power (1-β error probability) of 0.90, numerator degrees of freedom of 7, a total of 8 groups, and an effect size (f) of 0.5 which was derived from Partial Eta Squared (*η*^2^) values obtained during pilot testing. This resulted in a total sample size (*N*) of 81, which was rounded down to 80, with each group consisting of 10 samples (*n* = 10). This sample size is consistent with many studies assessing fracture strength of all ceramic and zirconia crowns (10, 26, 27).

The statistical analysis was carried out using IBM SPSS Statistics version 20 (IBM Corporation, Armonk, NY, USA). The normality of the data was evaluated with the Shapiro-Wilk test, which confirmed normal distribution across all groups (*P* values > 0.05). Univariate Analysis of Variance (Two-Way ANOVA) was conducted to assess the effects of material type, surface treatment, and the interaction of both factors on fracture load. An independent samples *T*-test was employed to evaluate differences between the different zirconia materials per each surface treatment group. One-Way ANOVA was performed to assess differences between the different treatment groups for each zirconia material. For detailed comparison between treatment groups for each material type, Tukey's *post hoc* test was used. Additionally, a Chi-square test was conducted to evaluate differences in fracture types among the different treatment and material groups. All statistical tests were performed with a significance level set at 0.05.

## 3 Results

Univariate Analysis of Variance (Table 2) showed a significant effect of material and surface treatment on fracture load (*P* < 0.001). The interaction effect between the two factors on fracture load was also significant (*P* = 0.004) suggesting that the effect of surface treatment on fracture load varied depending on the material used. An independent samples *T*-test revealed that the fracture load of 3Y-TZP (583.13–1109.07 N) was significantly higher than that of 5Y-PSZ (419.64–711.73 N) across all groups (*P* ≤ 0.001). One-Way ANOVA indicated significant differences between the various treatment groups for both materials (*P* ≤ 0.001). For both materials, the lowest fracture load was observed in Group 1, while the highest was in Group 4.

**Table 2:** Univariate Analysis of Variance (Two-Way ANOVA) test assessing the effect of the factors of material and surface treatment and their interaction on fracture load.

Source	Type III sum of squares	df	Mean square	F	Sig.	Partial eta squared
Corrected Model	3936670.727^a^	7	562381.5	44.766	<0.001	0.813
Intercept	37,764,033	1	37,764,033	3006.054	<0.001	0.977
Material	2,048,717	1	2,048,717	163.08	<0.001	0.694
Surface Treatment	1,706,940	3	568980.1	45.291	<0.001	0.654
Material * Surface Treatment	181013.4	3	60337.81	4.803	0.004	0.167
Error	904511.5	72	12562.66			
Total	42,605,215	80				
Corrected Total	4,841,182	79				

^a^ R Squared = .813 (Adjusted R Squared = .795).

In the case of 3Y-TZP, the fracture load in Group 2 (*P* = 0.002) and Group 3 (*P* < 0.001) was significantly higher compared to Group 1, showing an increase of 40% and 50% respectively. No significant difference was found between Group 2 and Group 3 (*P* = 0.755). Group 4 exhibited a significantly higher fracture load compared to Group 1 (*P* < 0.001), Group 2 (*P* < 0.001), and Group 3 (*P* = 0.003), with a 90% increase compared to Group 1.

For 5Y-PSZ, the fracture load increased by 18% in Group 2% and 15% in Group 3 compared to Group 1; however, these differences were not statistically significant (*P* ≥ 0.211). In contrast, Group 4 demonstrated a 70% increase in fracture load compared to Group 1, which was significantly higher than all other groups (*P* < 0.001).

**Figure 2:**
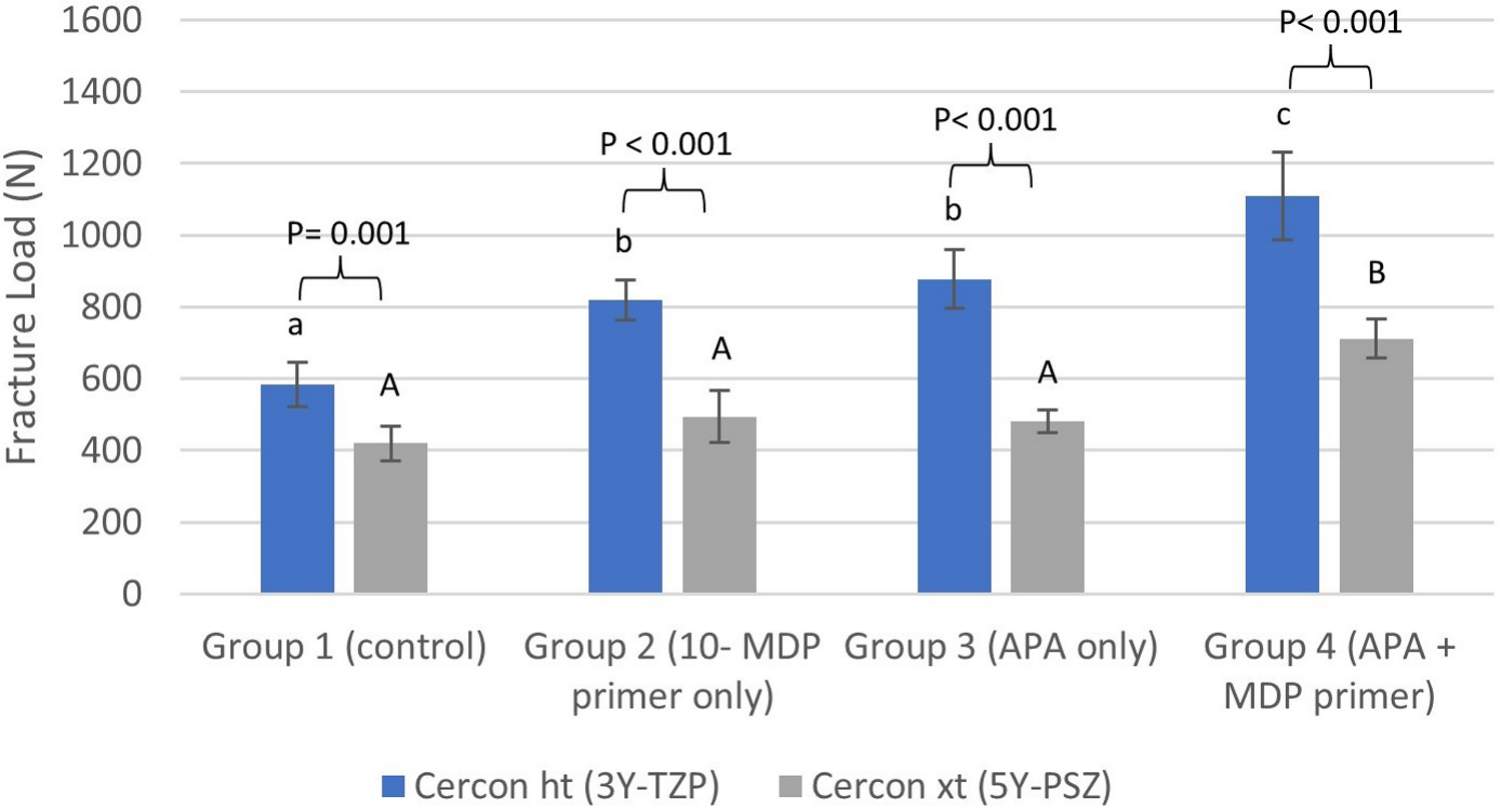
Bar chart illustrating the mean fracture load values in Newton **(*N*)** for different treatment groups for each material. Error bars represent two times the standard error (2SE). Letters on bars indicate statistically significant group differences within each material type based on Tukey's *post hoc* test: lowercase letters are used for Cercon ht (3Y-TZP), and uppercase letters are used for Cercon xt (5Y-PSZ). Different letters denote significant differences within each material type (*P* < 0.05). *P* values above brackets show the significance of comparisons between corresponding treatment groups for Cercon ht and Cercon xt. 10-MDP stands for 10-methacryloyloxydecyl dihydrogen phosphate, and APA stands for airborne-particle abrasion.

Table 3 summarizes the fracture load data for all groups, and Figure 2 illustrates the data for both materials across all groups.

**Table 3:** Mean values and standard deviation (in parentheses) of the fracture load of each treatment group per each material expressed in Newton. Values with the same superscript letters per column represent non-significant differences as assessed by Tukey *post hoc* test. The last row shows *P* values obtained from One-way ANOVA test. The last column shows *P* values obtained from independent samples *T*-test comparing values of each material in each treatment group (*α* = 0.05).

Treatment groups	Material		*P* value
	Cercon ht (3Y-TZP)	Cercon xt (5Y-PSZ)	
Group 1 (control)	583.13 (97.24)^a^	419.64 (75.32)^a^	**0** **.** **001**
Group 2 (10-MDP Primer^1^ Only)	818.25 (88.47)^b^	494.43 (113.09)^a^	**<0** **.** **001**
Group 3 (APA^2^ Only)	877.89 (130.78)^b^	482.32 (50.24)^a^	**<0** **.** **001**
Group 4 (10-MDP Primer + APA)	1109.07 (194.49)^c^	711.73 (85.45)^b^	**<0** **.** **001**
***P* value**	**<0**.**001**	**<0**.**001**	

^1^ 10-methacryloxydecyl dihydrogen phosphate.

^2^ Airborne-particle abrasion.

Regarding the type of fracture, Chi -square testing indicated no significant differences among the different treatment groups in 3Y-TZP [*χ*^2^ (12) = 9.189, *P* = 0.687] or 5Y-PSZ [*χ*^2^ (9) = 12.444, *P* = 0.189]. Additionally, there was no significant difference between the two materials concerning the type of fracture [*χ*^2^ (4) = 1.154, *P* = 0.886]. The predominant type of fracture was Type III (Table 4).

**Table 4:** Frequency and percentage (in parentheses) of fracture types across different treatment groups and the total frequency for each material. The last column displays *P* values from the Chi-square test comparing fracture type frequencies among treatment groups. The *P* value in the last row compares fracture type frequencies between the two materials (*α* = 0.05).

Material	Treatment group	Type of fracture					*P* value
		Type I	Type II	Type III	Type IV	Type V	
Cercon ht (3Y-TZP)^a^	Group 1 (control)	0 (0.0)	0 (0.0)	6 (60.0)	2 (20.0)	2 (20.0)	0.687
	Group 2 (10-MDP^b^ Primer Only)	0 (0.0)	1 (10.0)	5 (50.0)	2 (20.0)	2 (20.0)	
	Group 3 (APA^c^ Only)	1 (10.0)	0 (0.0)	8 (80.0)	1 (10.0)	0 (0.0)	
	Group 4 (10-MDP Primer + APA)	0 (0.0)	0 (0.0)	6 (60.0)	2 (20.0)	2 (20.0)	
	**Total**	**1** **(2.5)**	**1** **(2.5)**	**25** **(62.5)**	**7** **(17.5)**	**6** **(15.0)**	
Cercon xt (5Y-PSZ)^d^	Group 1 (control)	0 (0.0)	1 (10.0)	7 (70.0)	1 (10.0)	1 (10.0)	0.189
	Group 2 (10-MDP Primer Only)	0 (0.0)	0 (0.0)	10 (100.0)	0 (0.0)	0 (0.0)	
	Group 3 (APA Only)	0 (0.0)	0 (0.0)	6 (60.0)	2 (20.0)	2 (20.0)	
	Group 4 (10-MDP Primer + APA)	0 (0.0)	0 (0.0)	4 (40.0)	3 (30.0)	3 (30.0)	
	**Total**	**0** **(0.0)**	**1** **(2.5)**	**27** **(67.5)**	**6** **(15.0)**	**6** **(15.0)**	
***P* value**	**0** **.** **886**						

^a^ 3 mol% yttria-stabilized tetragonal zirconia polycrystals.

^b^ 10-methacryloxydecyl dihydrogen phosphate.

^c^ Airborne-particle abrasion.

^d^ 5 mol% yttria partially stabilized zirconia.

## 4 Discussion

The results showed that the type of zirconia material significantly affected fracture load; thus the first null hypothesis was rejected. In addition, the results showed that the use of 10-MDP primer and APA surface treatments either individually or in combination had a significant effect on the fracture load of the crowns thus the second null hypothesis was rejected.

The current study showed that the fracture load of 5Y-PSZ in all groups is significantly lower than 3Y-TZP. This is mainly due to the inherent flexural strength of the different materials where 3Y-TZP zirconia is characterized by higher flexural strength compared to 5Y-PSZ zirconia (28). This is very important to consider when selecting crown materials for the posterior teeth. The results of the current study showed that the incorporation of 10-MDP in the resin cement is not a substitute for the use of a 10-MDP primer in increasing the fracture load of zirconia crowns which aligns with findings from a previous study (21).

The effects of different surface treatments on fracture load observed in this study align with the impact of various surface treatments on bond strength reported in previous research (29). Significantly higher shear bond strength was achieved by combining sandblasting with etching systems and primers compared to sandblasting alone, corroborating the fracture load results in the current study. This indicates that bond strength is linked to fracture load and the survival of bonded zirconia restorations, similar to observations with glass ceramics (14). The creation of an integrated adhesive structure likely improved the fracture load by allowing the cement to absorb elastic stress and offset the rigidity of the zirconia core. This could reinforce the restorative system, dissipating occlusal loads across the entire intaglio surface of the crown. The use of bonding techniques compared to cementation has been shown to positively affect the fracture load of thin 3Y-TZP zirconia crowns. In a previous study, 0.5 mm and 0.2 mm thin 3Y-TZP crowns were tested, showing fracture loads of 1,628 N (SD = 174) and 1,357 N (SD = 340) for 0.5 mm crowns and 1,164 N (SD = 334) and 772 N (SD = 148) for 0.2 mm crowns, for adhesive bonding and cementation respectively (8).

The findings are further corroborated by comparing the results of the current study with those of a previous research where 0.5 mm thick 3Y-TZP and 5Y-PSZ zirconia crowns were air abraded only and cemented using resin-modified glass ionomer cement (10). In that study, the fracture load of 3Y-TZP zirconia crowns was higher than that of Groups 1 (control) and 2 (10-MDP primer only) in the current study, comparable to Group 3 (APA only), but lower than Group 4 (APA + 10-MDP primer). Regarding 5Y-PSZ zirconia, the fracture load was lower in that study compared to all groups in the current study. Thus, to enhance the fracture load of 0.5 mm 5Y-PSZ zirconia crowns, it is recommended to use self-adhesive resin cement in combination with both APA and 10-MDP primer for the best results.

In the current study, 3Y-TZP demonstrated significant benefits when the 10-MDP primer was applied individually, showing a 40% increase in fracture load despite the presence of the 10-MDP monomer in the resin cement used for the control. This improvement can be attributed to the lower viscosity and higher concentration of the 10-MDP monomer in the priming agent compared to the resin cement, allowing for better flow over the zirconia surface and stronger chemical interaction (21). Additionally, 3Y-TZP benefited from air abrasion alone, exhibiting a 50% increase in fracture load compared to the control. Conversely, 5Y-PSZ did not benefit from either APA or the 10-MDP primer when used individually.

The differences in fracture load between 3Y-TZP and 5Y-PSZ after APA treatment is primarily due to the different compositions of the materials. APA has been shown to trigger a tetragonal-to-monoclinic phase transformation in conventional zirconia, such as 3Y-TZP, as demonstrated by µRaman spectroscopy and x-ray diffraction analysis (22, 25, 30). The impact of the abrasive particles induces this transformation due to localized stresses, generating volume expansion (3%–5%) and compressive residual stresses on the surface of 3Y-TZP. These compressive stresses counteract crack propagation, enhancing the flexural strength and fracture toughness of the material (22, 25, 30), which likely contributed to the increased fracture load of 3Y-TZP in the current study. In contrast, 5Y-PSZ did not benefit from APA alone due to its higher content of cubic grains, which have less potential for phase transformation into the monoclinic phase and, consequently, less transformation toughening (17, 31). Although APA alone may increase surface free energy and wettability by decontaminating the surface and increasing roughness, enhancing bond strength (16), this effect is diminished in 5Y-PSZ because the material does not experience phase transformation toughening. Microcracks resulting from air abrasion may not be adequately counteracted in 5Y-PSZ zirconia, especially at pressures greater than 0.2 MPa (23).

Regarding the effect of 10-MDP primer, 3Y-TZP may have higher surface free energy and chemical affinity compared to 5Y-PSZ due to its higher tetragonal zirconium oxide content, providing a more wettable surface and better interaction with the 10-MDP monomer, resulting in stronger bonding and, consequently, greater fracture load. In contrast, 5Y-PSZ did not show similar benefits from the 10-MDP primer alone.

Both materials benefitted significantly when APA and the 10-MDP primer were applied simultaneously, with a 90% increase in fracture load for 3Y-TZP and a 70% increase for 5Y-PSZ, demonstrating a synergistic effect. The greater increase in 3Y-TZP compared to 5Y-PSZ is again could be to the combined benefits of transformation toughening and increased chemical bond strength. This is supported by previous research, which found that the shear bond strength of 3Y-TZP tended to be higher than that of 5Y-PSZ when both materials were air abraded and treated with a 10-MDP universal primer, especially after prolonged water storage (32).

The peak bite forces in the oral cavity are influenced by factors such as age, gender, craniofacial structure, the status of the temporomandibular joint, occlusal characteristics, and the specific location within the mouth where the measurement is taken. Generally, posterior teeth endure greater bite forces compared to anterior teeth (33). Research has shown that the bite forces in the molar regions typically remain below 900 N (34). In a previous research, the maximum voluntary bite force (MVBF) recorded was 1642.8 N, and the minimum was 83.9 N. The mean MVBF was 430.4 N (SD = 279.4). Specifically, in the molar region, the MVBF was higher, with an average of 493.4 N (SD = 32.5), compared to the premolar region, which had an average MVBF of 383.3 N (SD = 22.8) (35). Therefore, it is advised that restorations should be able to withstand bite forces exceeding 500 N in the challenging conditions of the oral environment (36). Clenching may lead to occlusal loads between 500 and 800 N, and in severe cases even up to 1,000 N (37). Based on the data from Table 3 and the provided information on maximum bite forces, it is recommended to use 0.5 mm thickness 3Y-TZP for restorations in high-stress areas such as the molar regions, specifically, 3Y-TZP treated with APA and 10-MDP primer together (Group 4) which offers the highest mean value of 1109.07 N, making it ideal for regions subjected to severe occlusal loads and clenching forces. For posterior teeth, 3Y-TZP in Groups 2 and 3, which also show significant fracture load (818.25 N and 877.89 N respectively), are suitable. 5Y-PSZ zirconia, particularly when treated with APA and 10-MDP primer (Group 4), with a mean fracture load value of 711.73 N can be recommended for anterior and premolar regions. While 5Y-PSZ in Groups 1, 2, and 3 shows lower fracture load values, it can still be considered for less demanding applications. Overall, restorations should be capable of withstanding at least 500 N, with higher thresholds preferred for posterior teeth to ensure durability and longevity in the hostile oral environment.

The main limitation of the current study is that it is an *in vitro* study in which only static loading was applied. *in vitro* studies, while beneficial for their controlled environments and reproducibility, fail to capture the complex biological and functional dynamics of the oral environment, such as interactions with saliva and varied biting forces, which can significantly impact the real-world performance of a restorative materials. They also fall short in predicting long-term outcomes. Thus, the results of the current study although give insight into the mechanical performance of the different materials and different bonding conditions, these results should be considered carefully. Accordingly, comprehensive clinical studies, which assess thin zirconia crowns under actual functional conditions are recommended to provide conclusions regarding their success rate and longevity.

Within the limitations of this study, the following conclusions can be drawn: thin zirconia crowns (0.5 mm thickness) fabricated from 3Y-TZP exhibit higher fracture load values compared to those made from 5Y-PSZ. For 3Y-TZP, the application of a 10-MDP primer or airborne-particle abrasion (APA) significantly increases the fracture load, with the combination of both treatments yielding the highest values. In the case of 5Y-PSZ, a significant increase in fracture load is observed only when both APA and the 10-MDP primer are used together. These findings reinforce the traditional protocol for zirconia cementation, which includes the use of both APA and 10-MDP primer, particularly for the new generation 5Y-PSZ zirconia, as it is crucial for enhancing fracture load in high-stress situations.

## Data Availability

The raw data supporting the conclusions of this article will be made available by the authors, without undue reservation.
